# Effects of Language Proficiency on Selective Attention Patterns at Segmenting Boundaries in English Audio Sentences

**DOI:** 10.3390/brainsci14121204

**Published:** 2024-11-28

**Authors:** Yunhao Mei, Fei Chen, Xiaoxiang Chen

**Affiliations:** School of Foreign Languages, Hunan University, Lushannan Road No. 2, Yuelu District, Changsha 410082, China; michaelyh@hnu.edu.cn (Y.M.); chen630610@aliyun.com (X.C.)

**Keywords:** language proficiency, segmentation boundaries, selective attention pattern, memory

## Abstract

Background/Objectives: Normative perceptual segmentation facilitates event perception, comprehension, and memory. Given that native English listeners’ normative perceptual segmentation of English speech streams coexists with a highly selective attention pattern at segmentation boundaries, it is significant to test whether Chinese learners of English have a different attention pattern at boundaries, thereby checking whether they perform a normative segmentation. Methods: Thirty Chinese learners of English with relatively higher language proficiency (CLH) and 26 with relatively lower language proficiency (CLL) listened to a series of English audio sentences. Meanwhile, they were asked to press the key whenever a phonetic probe “ba” occurred. Response time to “ba” reflects the attention where “ba” is located at segmentation boundaries. Results: The results revealed that, (1) relative to native English listeners’ highly selective attention pattern, the CLH group showed a relatively selective attention pattern, while the CLL group displayed a non-selective attention pattern. (2) Both the CLH group and natives had better recognition memory than the CLL group. (3) Both the CLH group and natives’ attention at segmentation boundaries was not correlated with their memory for sentences, while the CLL group’s attention at boundaries was correlated with memory. Conclusions: These findings suggest that (1) Chinese learners of English did not perform a normative segmentation, which shows the effect of English proficiency on perceptual segmentation; (2) English proficiency has a superior effect on memory for sentences, while perceptual segmentation would come next to support memory by providing structure for memory construction if English proficiency is not high; (3) a comparison of attention patterns between Chinese learners and natives can provide a reference for potential intervention to rectify non-natives, thereby improving their perception of English speech streams.

## 1. Introduction

People are inclined to segment the (seemingly) continuous world into lots of discrete events, such as go shopping, make breakfast, etc. Likewise, people tend to divide languages into discrete units, such as sentences, phrases, words, syllables, and phonemes, which have been treated as different language events [1,2,3]. Event segmentation theory (EST) [4,5] explains how events are segmented, proposing that knowledge and sensory features are two key factors to influence event segmentation. However, findings about the effect of knowledge on the segmentation of events are not consistent. Thus, it is necessary to further investigate the effect of knowledge on the event segmentation. As mentioned above, sentences are one specific event in language, while language proficiency reflects one’s particular knowledge about language. Thus, it may be possible to investigate the effect of knowledge on the event segmentation by exploring the role of language proficiency in the continuous speech segmentation.

Native English listeners’ normative perceptual segmentation of English audio sentences coexists with a highly selective attention pattern at segmentation boundaries [6]. Thus, this raises a question as to whether non-natives also display the same attention pattern at segmentation boundaries as the native. If they really have the same pattern as the native, it would imply that non-natives have performed a normative perceptual segmentation like the native; on the contrary, if they do not, it might indicate that non-natives have not made a normative segmentation like the native. According to the proposed role of knowledge in perceptual segmentation by the EST [4,5], it is hypothesized that non-natives with lower language proficiency should not make a proper perceptual segmentation of English audio sentences. That is, non-natives’ attention pattern at segmentation boundaries is hypothesized to be different from that of the native English listeners. Therefore, the first aim of this study is to investigate whether Chinese learners of English have a normative perceptual segmentation by comparing their attention patterns at boundaries with that of the native English listeners.

Previous studies show that knowledge facilitates event memory by providing knowledge structures for memory construction and retrieval structures for memory retrieval [7,8,9]. Prior research also testifies that perceptual segmentation aids memory for events by endowing the segmentation structure, i.e., hierarchical structure of fine-grained segmentation enclosed by coarse-grained segmentation, and temporally sequential structure [10,11]. According to the EST [4,5], knowledge can enhance perceptual segmentation. Therefore, it gives rise to a second question: what is the relationship among knowledge, perceptual segmentation, and memory? In other words, how do knowledge and perceptual segmentation affect memory specifically? Based on previous relevant empirical findings (listed in the last part of the Introduction), it is hypothesized that knowledge would have a dominant role in affecting later memory; however, when knowledge does not exist or is insufficient, perceptual segmentation would come to help support memory. Therefore, the second aim of this study is to disentangle the relationship of the effect of knowledge and perceptual segmentation on memory by taking all three factors together in consideration.

### 1.1. Event Segmentation Theory

In the past, many theories have been proposed to deal with event cognition, but with emphases on different areas: some focus on perception [12,13,14], others are concerned with memory and inference [15], and still others are interested in action control [16]. Some current theories try to combine some of those above areas. For example, the Theory of Event Coding (TEC) [17] posits that perception and actions have shared representations [4]. In addition, the REtrospective and PRospective Inference SchEme (REPRISE) model [18] is a recurrent neural network, which can learn to infer and identify vehicle changes (event segmentation) and drive the vehicle [4]. REPRISE shows the key role of modeling temporal structure in activity whose mechanisms have been discussed by the EST [4,5].

Event segmentation theory [4,5] deals with how people segment ongoing activities into discrete events from a mind–brain perspective. The term “event” is defined as a segment of time conceived by an observer, which has a beginning and an end [3,5]. According to Zacks and Tversky [3], event units quantitatively resemble phonemes, syllables, words, etc., in language. Thus, the language-specific segmentation in this study is also quantitatively analyzed using the EST.

According to the EST [4,5], people first take a representation of the current world, including perceptual information, language, and other sources and make predictions about what will happen near in the future. In the meantime, event models (i.e., working memory representations about ‘what is happening now’) are constructed, which will guide people to predict the incoming information. As the established event models fit with the real world, perceptual processing is facilitated. The periods when event models are maintained form events. Event model maintenance depends on the medial prefrontal and posterior cortex and parts of the lateral inferior parietal cortex [4,19]. As time goes on, however, when event models are no longer fitted into the outside world, prediction errors will transiently increase, and event model updating occurs (so as to appropriately represent the world). This error-based updating mechanism is carried out by the midbrain dopamine system’s phasic activity [20]. The time periods when event models update are event boundaries (segmenting two adjacent different events), thus accomplishing event segmentation.

The EST [4,5] claims that event segmentation is a spontaneous concomitant of perception for everyday activities, which is affected by knowledge and changes of sensory features. Knowledge here refers to all kinds of top-down factors, such as language proficiency [1,21], knowledge [4], prior knowledge [5], expertise [22,23], perspective [24,25], familiarity [26,27,28], goals and causal relations [23,29,30], context [31,32], culture [33], etc. Particularly, given that non-native listeners in the previous studies of language segmentation are either well able to understand or totally unable to speak and understand their second language, the study on the effect of non-natives’ different levels of language proficiency on their segmentation behavior can fill the gap between event cognition and second language learning research [1]. Event segmentation is asserted to be a core domain-general mechanism of cognitive control to modulate attention and memory, which is sub-served by specific neural mechanisms [5,20,34]. For example, it is testified that people tend to remember information at event boundaries better than non-boundaries, called the event boundary advantage effect (the EBA effect) [30,35,36,37]. The EST considers the EBA effect to be that along with event segmentation, transiently increased prediction errors and event model updating at event boundaries attract much more attention to information at event boundaries than non-boundaries, finally leading to superior memory for information at event boundaries [35,37].

### 1.2. Effects of Knowledge on Event Segmentation

As for the effect of knowledge on event segmentation, there are currently two lines of research. One is about whether knowledge plays a necessary role in event segmentation, i.e., whether event segmentation can be performed even if there is no knowledge. The other is whether knowledge plays a role in event segmentation if knowledge exists. That is to say, if knowledge is added, will there be any influence on event segmentation. The study of Hard et al. [27] belonged to the former, which asserted that event segmentation was purely dependent on changes in sensory features, which could be performed even without knowledge. In regard to top-down knowledge, it mainly affects the after-the-segmentation interpretation [27]. Through a series of experiments, Feller [22] also showed that event segmentation was in fact influenced by physical changes, and knowledge made an impact on the downstream processing (after the segmentation), such as the event ratings and descriptions of events. In other words, changes in sensory features are sufficient for the segmentation of events, and knowledge is not necessary in event segmentation. Taking another example, Huff et al. [35] found that fandom only biased retrospective judgments but not event segmentation, indicating that top-down factors of attitudes and motivations yielded from fandom do not influence the initial stage of processing.

However, there is also criticism for Hard et al. [27]’s studies because they usually use simple material and tasks, which is the reason why knowledge seems not necessary for event segmentation. For example, Hard et al. [27] asked participants to segment geometric figures in motion, which is much simpler than real-life events or language events. It is important to note that though Hard et al. [27] claimed knowledge did not play a necessary role in segmentation, they still agreed that knowledge indeed played a role in event segmentation if knowledge was manipulated. For example, the number of event segmentation sequences becomes smaller if participants are familiar with the experimental material beforehand [27].

Actually, lots of other studies also give their support for the role of knowledge in segmentation of events by the manipulation of knowledge. For example, basketball experts show higher segmentation agreement at the coarse level relative to novices [38]. Consistent with that, figure skating experts also present higher segmentation agreement for coarse sub-events than control novices [23]. Furthermore, Goldstone [39] affirmed that expertise-related optimization of perceptual processes occurred very early in the stream of information processing, which further influenced all of the following processes [40]. The experts’ long-term perceptual learning yields changes, oftentimes strikingly large, to perceptual systems through mechanisms of attentional weighting, stimulus imprinting, differentiation, and unitization [39]. In other words, expertise can not only influence but also permanently reshape the early perceptual processing of stimuli in their expertise domain.

Apart from expertise, goals and causal relations are testified to influence event segmentation [28,29]. The Event Indexing Model [41] put forward five dimensions to be used for narrative segmentation, i.e., time, space, characters, intention, and causes. Evidence reveals that infants are more surprised, or with longer viewing time when a pause is inserted into event middles than event boundaries of a goal-directed activity [42,43]. In addition, culture also affects event segmentation by the modulation of attention to different aspects of activities [33]. In observing coffee-making activities, US participants identifying event boundaries is more correlated with visual changes, while the Indian’s event segmentation is more associated with goal changes [33].

Language proficiency is also revealed to affect the segmentation of language events. For example, Gilbert et al. [21] testified the effect of language proficiency on phrase parsing in a second language. They found that a higher language proficiency level was required for French learners of English to parse English utterances in an English-native-like manner. Furthermore, other forms of knowledge have also been shown to impact event segmentation, such as contextual knowledge [32,44] and background knowledge [45,46], life knowledge [47], etc.

Nevertheless, the effect of knowledge on segmentation is not affirmed by all studies. For example, as for the effect of perspectives on event segmentation, Newberry and Bailey [32] instructed participants to read and segment passages either in the perspective of a burglar or a home buyer. Results disclosed that different perspectives affected segmentation agreement among participants. Nevertheless, there are contrary findings as for the effect of perspectives. For instance, Magliano et al. [24] and Swallow et al. [25] showed that participants had the same good segmentation agreement whether in the first-person or third-person perspective. Additionally, the impact of familiarity on event segmentation is not totally agreed upon. Some studies approve the effect of familiarity on event segmentation [48,49], while others do not [28,50]. Therefore, the effect of knowledge on event segmentation cannot be taken for granted, requiring further exploration.

As seen above, some kinds of knowledge seem to influence event segmentation, while others do not; some forms of knowledge seem to have consistent findings on their effect on event segmentation, while others have an inconsistent effect. As for the reasons for such different and inconsistent findings regarding the effect of knowledge on event segmentation, to our knowledge, there has not been relevant literature specific to discussing it. Firstly, it may be that these different forms of knowledge have different amounts of top-down influence on people’s perceptual system. Specifically, if the form of knowledge is the result of long-term accumulation (such as expertise, culture, background, and life knowledge) or permanent habituation due to the evolution of human beings (such as goals, causal relations, and contextual knowledge), it has reshaped people’s perceptual system, just as Goldstone [39] said, and is, thus, strong enough to exert a stable effect on event segmentation. However, if the type of knowledge is achieved from short-term experience (such as familiarity) or for temporal use (such as perspectives), it has not yet produced stable changes for one’s perceptual system, thereby relatively weak in supporting a steady influence on event segmentation. Secondly, it may depend on the grain of segmentation about whether the effect of knowledge can be observed. Research has shown that as the time scale of segmentation increases (i.e., from fine-grained to coarse-grain segmentation), events are more determined by goals, intentions, etc., than physical features [3,28,51], displaying the role of knowledge.

### 1.3. Effects of Knowledge and Event Segmentation on Subsequent Memory for Events

Earlier studies show that more knowledge might lead to better memory for events [7,8,9]. For example, both Chase and Ericsson [7] and Ericsson and Kintsch [8] assumed the importance of retrieval structures developed by experts in facilitating memory. In other words, experts with more domain knowledge are able to develop memory skills to improve memory.

Meanwhile, prior studies also reveal that better (more normative) event segmentation results in better subsequent event memory [10,11,49,52]. For instance, Gold et al. [52] asked participants to watch and remember movies whose natural event boundaries were placed with auditory or visual cues. The results showed that movies with boundary cues were remembered better than those without boundary cues, indicating the effect of event segmentation on memory for events. Flores et al. [10] further found that the facilitating role of event segmentation in memory can last over a long delay, up to one month. Moreover, the individual difference in event segmentation might be related to the individual difference in memory over long delays [10].

Interestingly, as introduced above, studies also show that increased knowledge causes better event segmentation [4,5,21,23,29,38]. Then, there is a question whether it is through better event segmentation that knowledge facilitates subsequent memory. Actually, a few previous studies tried to resolve this problem, but agreement has not been reached. Some assert that knowledge indeed affects later memory through event segmentation [32,53,54,55], while others do not [4,28,56]). For example, Baldassano et al. [53] claimed that the brain had anticipatory reinstatement of event patterns (event segmentation) when listening to a familiar narrative (i.e., with prior knowledge), which is the basis for subsequent memory. That is to say, prior knowledge has an effect on the perceptual segmentation first, and then influences memory representations. Newberry and Bailey [32] also considered that (contextual) knowledge first played a role in event segmentation, which then affected later memory for texts, though they also inferred that event segmentation was probably one of several possible means (e.g., mental imagery, elaboration) by which (contextual) knowledge influenced later memory.

However, Sargent et al. [56] supported that knowledge affected memory independently, because they revealed that knowledge and event segmentation independently predicted subsequent memory. Just like what Zacks et al. [28] said, it was possible that conceptual knowledge influenced memory though the reconstruction of events during the period of retrieval rather than the early period of encoding, that is, not making an impact on the online event segmentation at all [57,58].

In addition, another view worthy of note is that knowledge affects memory through the reconstruction of events only if relevant knowledge is available; at this time, event segmentation influences memory when one has little knowledge to rely on [30,38]. Thus, it is necessary to further explore how knowledge and event segmentation impact event memory.

## 2. Methodology

According to the attentional boost effect observed in auditory oddball detection, where transient increases in attention to one task enhance performance in a second task [59], transient increases in attention to audio sentence processing are expected to improve the detection of an embedded attention probe, that is, faster detection of the attention probe within the auditory sentence. In other words, response times (RT) for the attention probe embedded in the audio sentence reflects the attention fluctuation along with audio sentence processing: less RT for the probe indicates more attention to the place where the probe is located (in the audio sentence processing), and more RT for the probe reflects less attention to sentence processing at that time. For simplicity, RT for the attention probe negatively reflects the attention therein [60,61].

Language proficiency mirrors people’s knowledge about the language and ability or skills to use that knowledge, including listening, speaking, reading, writing etc., which belongs to top-down conceptual knowledge. In view that it is the English language itself used in this study, foreign language learners’ different language proficiency levels reflect their knowledge about English. Two groups of participants, i.e., Chinese learners of English with relatively higher language proficiency (CLH) and Chinese learners of English with relatively lower language proficiency (CLL), were instructed to listen to a series of isolated audio sentences and press the space bar on the computer keyboard as quickly as possible whenever they heard a sound of “ba” (the attention probe) embedded in the audio sentences. RT was logged and compared across the three temporal locations around event boundaries: the event beginning, the event segmentation point, and the event ending. Additionally, recognition accuracy (memory) for these sentences was calculated.

### 2.1. Participants

A priori power analysis in the chi-square goodness-of-fit test (parameters: effect size w = 0.5; α error probability = 0.05; power (1 − β error probability) = 0.95; degrees of freedom = 1) was performed by using the G*Power (version 3.1.9.7) [62,63]; a minimum sample size of 52 participants was required for this experiment. We invited 30 CLH listeners (21 females; *M*age = 24.5 years, *SD* = 1.12) with an age range from 22 to 26 years old, and 26 CLL listeners (15 females; *M*age = 19.3 years, *SD* = 0.83) with an age range from 18 to 22 years old. There were 26 native English listeners (the NE group; 16 females, 10 males; *M*age = 24.8 years, *SD* = 2.45; age range: 19 to 29 years’ old) in the prior study [6].

The CLH listeners were Chinese university students who are English majors, and they had passed the Test for English Majors Band-8 (TEM-8; the highest-level national test for senior-undergraduates English majors in China). The CLL participants were Chinese university students who are not English majors, and they had not yet participated in the College English Test Band-4 (CET-4; a relatively lower-level national standardized English language test for all non-English-major college and university students in China). None of the CLH and CLL participants had any hearing impairment. They were all right-handed. All participants signed written informed consent and received monetary compensation for their participation. This research was approved by the Research Ethics Committee of School of Foreign Languages, Hunan University (protocol code SOF20210311).

### 2.2. Materials

The same experimental materials in the prior study to explore the NE group’s attention pattern at event boundaries [6] (available at https://osf.io/zk58a/, accessed on accessed on 20 November 2024) were used in this experiment. So, here, we briefly introduce the experiment materials. We chose and adapted 50 English sentences from the TIMIT corpus [64]. A native English-speaking young woman from New York was asked to read these sentences naturally at a normal rate. Audacity (version 3.0.2, https://audacity.sourceforge.net/, accessed on 15 April 2021) was used to record all the sentences into WAV files at 16-bit mono with a sampling rate of 44,100 Hz.

Three native speakers of English were asked to listen to the recorded English audio sentences and segment each sentence into the largest units, which seemed to them natural and meaningful, so as to identify event boundaries [36,65,66,67]. For each sentence, only when all of the boundaries in the sentence (which were independently marked by the three native speakers) were identical was the sentence approved for experimental use. Thus, 30 audio sentences were approved. After that, these 30 sentences were screened by two English-speaking Americans to see whether they are accepted or commonly used in their daily life. Finally, 15 of the 30 spoken sentences survived the formal experiment. The 15 experimental auditory sentences have a mean duration of 4.32 s (*SD* = 0.70 s). A syllable “ba” was recorded by asking the same American young women who read the original sentences. Using Praat (version 6.1.50, http://www.praat.org/, accessed on 10 February 2021), the syllable “ba” was normalized to be a length of 0.2 s (with 189 Hz and 65 dB SPL being equal to the mean values of all experiment sentences).

**Figure 1:**
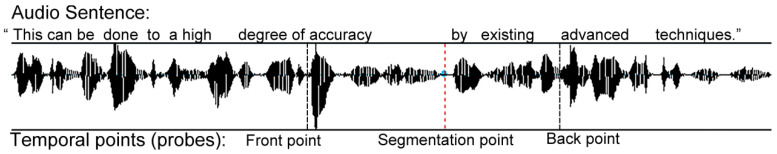
Schematic illustration of the three versions of each experimental sentence.

Each audio sentence had three versions (Figure 1): the front point version, the segmentation point version, and the back point version. In the front point version, an attention probe “ba” was embedded at the front point, which was two to four syllables before the segmentation point. In the segmentation point version, the attention probe was embedded at the segmentation point. In the back point version, the attention probe was embedded at the back point, which was two to four syllables after the segmentation point. It is worthy of note that the sound “ba” replaced the speech content at the front, segmentation, and back points, which means that a little speech was missing at those time points. However, this should not be a concern, because minor omissions are unlikely to alter the underlying predictability parameters of an ongoing event [66]. Therefore, attentional profiles related to event segmentation are robust to missing information at event boundaries.

The temporal range covering the three points (i.e., the temporal distance from the front point all the way to the back point) was around one second (*M* = 1.05 s; *SD* = 0.18 s). Each version had 15 target sentences.

In addition, segmentation boundaries in this experiment usually appear in the central or near-central parts along the audio sentence, so the attention probe accordingly frequently occurred in those temporal parts. To avoid that participants would always expect an occurrence of attention probe in some specific temporal points when hearing audio sentences, 17 filler sentences were added. For three of these filler sentences, the attention probe was embedded at the word boundary between the first and second words of the sentence; three other filler sentences were used with the attention probe being embedded at the word boundary between the last and penultimate words of the sentence; and the remaining 11 sentences were not embedded with any attention probe. This method of adding filler sentences has been testified in previous studies [61]. The experiment had three blocks, each of which consisted of 32 experiment sentences (15 target sentences and 17 filler sentences).

### 2.3. Procedures

The same procedures were adopted in this experiment as the previous study for the NE group [6]. Pilot studies had been conducted to validate the experimental design and materials. Before entering the formal experiment, participants were asked to familiarize the task using stimuli other than formal experimental ones.

The first author instructed participants to perform the experiment individually in a quiet room. The experiment was run by using a custom-made software package in PsychoPy (version 3.0, https://psychopy.org, accessed on 4 June 2021) [68] on a computer. Participants wore a wired earphone connected with the computer to hear the experimental stimuli. Participants were asked to complete two tasks: one was to listen to and remember the broadcast audio sentences; the other was to press the space bar on the keyboard as soon as possible whenever an incidental “ba” was heard in the perception of sentences. They were informed in advance that a subsequent memory test for the sentences would be taken afterwards. The three blocks of experiment trials were performed in a counter-balanced manner. In the follow-up recognition test, participants were instructed to identify whether these individually (visually) presented sentences in the computer were ever heard in the previous session, by pressing either A (‘yes’) or B (‘no’) on the keyboard. The visually presented sentences are either the verbatim original experimental sentences heard before (‘yes’ sentences) or other sentences never heard before (‘no’ sentences). These “yes” sentences are different from “no” sentences, no matter in the lexical or thematic sense. It would be logged as right (‘1’) if participants pressed the key “A” when they saw a visually presented “yes” sentence, or they pressed the key “B” when they saw a visually presented “no” sentence. On the contrary, it would be recorded to be wrong (‘0’) if participants did not make a congruent response.

Given that both the experimental procedures and stimuli in the current research are the same as those of the NE group (native English-speaking listeners) in the prior study, and the stimuli is in English, the NE group’s performances in the key-pressing and memory tasks are treated as control conditions.

### 2.4. Data Analyses

RT was transformed into inverse RT based on the Box-Cox procedure [69] to satisfy the normal distribution and homoscedasticity assumption for linear models, which was considered better than the simple logarithmic transformation [70]. In addition, the inverse RT transformation was also deemed best suited for psycho-linguistic data [71]. In view that this research is focused on cognitive and psychological processes in the perceptual processing of speech streams, the inverse RT was believed to be appropriate for this study.

Because this study aims to answer whether Chinese learners of English (the CLH and CLL groups) have displayed different attention patterns from that of the NE group, the RT value at each temporal point of segmentation boundaries for both the CLH and CLL groups should be compared to plot their relative position, i.e., the attention pattern. Thus, a linear mixed-effect model (LMM) was established to achieve the best fitting model predicting participants’ (inverse) RT by using statistical software R (version 4.0.5) [72] and the “lme4” package (version 1.1-7) [73]. To compare their recognition memory performance with that of the NE group, a full model (lmer(InverseRT~1+Location*Group+(1+Location|Subject)+(1+Group|Item), data)) was constructed with Location (the front, segmentation, and back points), Group (the NE, CLH, CLL groups), and their interaction as the fixed effect; with the by-participants and by-items random intercepts, the slope of by-participants for the Location and the slope of by-items for the Group as random effects were used to keep them maximal [74]. The final model (lmer(InverseRT~1+Location*Group+(1+Location|Subject)+(1|Item),data)) was achieved through the random-effect and fixed-effect trimmings. This method of establishing LMM models has been validated by previous studies [75].

The results from the recognition memory test were transferred as “0” (wrong) and “1” (right). Because they are binary data, generalized linear mixed-effect model (GLMM) was constructed to attain the best fitting model predicting participants’ recognition results. To compare their recognition memory performance with that of the NE group, a full model (glmer(Accuracy~1+Group+(1|Subject)+(1+Group|Item),family=binomial, data)) was established with Group (the NE, CLH, and CLL groups) as the fixed effect; the by-participant and by-item random intercepts, and the slope of by-item for the Group as random effects to keep it maximal [74]. The final model (glmer(Accuracy~1+Group+(1|Subject)+(1|Item),family=binomial, data) was obtained through the random effect trimming.

Finally, in view that both the RT at event boundaries and recognition accuracy are continuous variables, Pearson’s r correlation between the (inverse) RT at event boundaries (including the inverse RT at each specific point, and the summed inverse RT from all of the three points) and the recognition accuracy was performed (for each target sentence), so as to explore the relationship between the attention at event boundaries and the subsequent recognition memory. For comparison, the NE group’s correlation between the attention at event boundaries and the subsequent recognition memory was also calculated. All of the data and R scripts involving with RT, recognition accuracy, and their correlation mentioned above are available at https://osf.io/khtq4/.

## 3. Results

### 3.1. Selective Attention Patterns

Trials failing to detect the probe “ba” were trimmed from the data analysis (the CLH group: 1.3%; the CLL group: 1.8%). In addition, trials with RT less than 100 ms (false alarms) or more than 2500 ms (possible for sentence processing) [61,76] were excluded (the CLH group: 0.4%; the CLL group: 0.6%).

**Figure 2:**
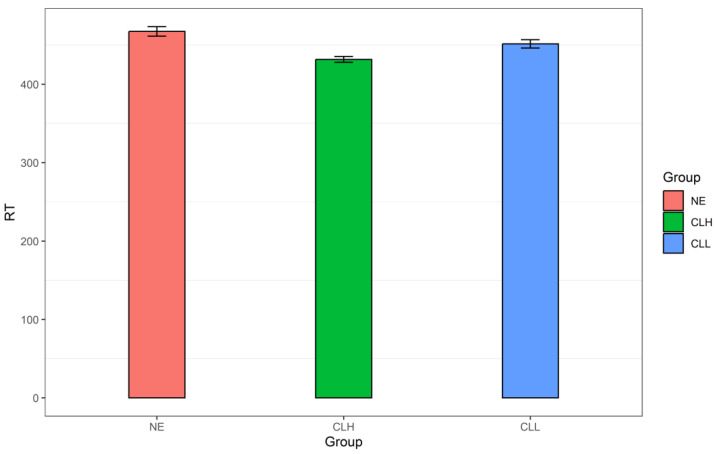
The three groups (the NE, CLH, and CLL groups)’ mean RT for the event boundary. Note. RT: response time; NE: the native English group; CLH: Chinese learners of English with relatively higher language proficiency; CLL: Chinese learners of English with relatively lower language proficiency.

As for the mean RT for the event boundary (i.e., across the front, segmentation, and back points), the CLH group triggered 432 ms (*SD* = 137 ms), and the CLL group had 452 ms (*SD* = 178 ms). For comparison, the NE group’s mean RT across the three points (*M* = 467 ms, *SD* = 195 ms) is also included here (see Figure 2).

The LMM on the (inverse) RT revealed a non-significant main effect of Group (χ^2^ (2) = 0.67, *p* = 0.716), a significant main effect of Location (χ^2^ (2) = 17.95, *p* < 0.001), and a marginally significant interaction between Group and Location (χ^2^ (4) = 8.46, *p* = 0.076). The non-significant main effect of Group suggests that there is no significant difference among the three groups’ mean RT at event boundaries. It should be of note that this lack of RT difference at event boundaries among the three groups only implies the overall amount of RT (or attention) at segmentation boundaries is the same. However, this study aims to see whether the CLH and CLL groups had a different attention pattern at boundaries from that of the NE group. That means that for each group, the RT values at the three time points should be compared so that the RT pattern can be plotted first. If the three groups’ plotted RT patterns are different from each other, it indicates that language proficiency really influences perceptual segmentation, or the CLH and CLL group did not perform a normative segmentation, because a proper segmentation should be with a highly selective attention pattern, just like the NE group [6]. In view of this, we made post hoc pairwise comparisons of the RT values across the three temporal locations for each group as follows.

**Figure 3:**
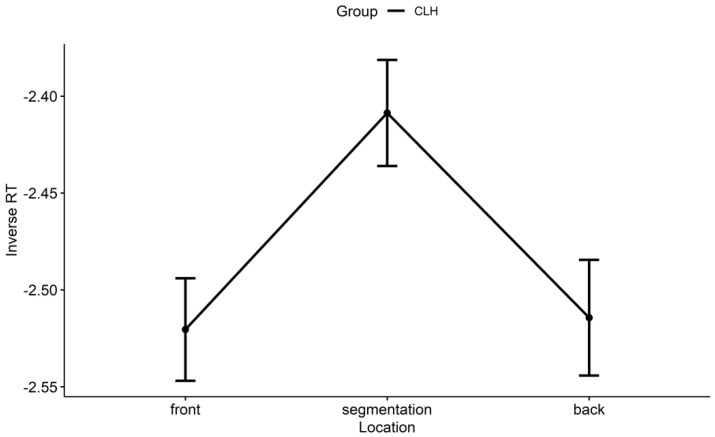
The CLH group’s (inverse) RT at the front, segmentation, and back points of event boundaries along audio sentences. Note. RT: response time; CLH: Chinese learners of English with relatively higher language proficiency.

**Figure 4:**
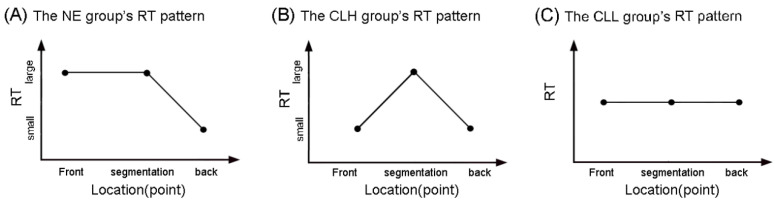
Schematic illustration of the NE, CLH, and CLL groups’ RT patterns at event boundaries. Note. RT: response time; NE: the native English group; CLH: Chinese learners of English with relatively higher language proficiency; CLL: Chinese learners of English with relatively lower language proficiency.

As seen in Figure 3, the CLH group had a significant (inverse) RT difference between the front and segmentation points (β = −0.12, *SE* = 0.04, *z* = −2.83, *p* = 0.013) and a significant (inverse) RT difference between the back and segmentation points (β = −0.11, *SE* = 0.04, *z* = −2.46, *p* = 0.037) but showed no reliable difference between the back and front points (β = 0.01, *SE* = 0.04, *z* = 0.19, *p* = 0.981). For parsimony, the CLH group’s RT pattern at event boundaries can be described as a small–large–small RT pattern, as shown in Figure 4B. Additionally, the NE group’s large–large–small RT pattern [6] is also contained in Figure 4A for comparison.

**Figure 5:**
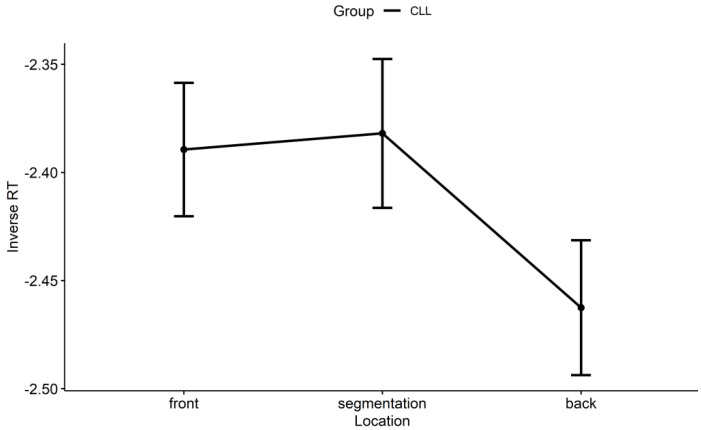
The CLL group’s (inverse) RT at the front, segmentation, and back points of event boundaries along audio sentences. Note. RT: response time; CLL: Chinese learners of English with relatively lower language proficiency.

In addition, as displayed in Figure 5, for the CLL group, there was no significant (inverse) RT difference between the front and segmentation points (β = −0.00, *SE* = 0.04, *z* = −0.07, *p* = 0.997), no significant (inverse) RT difference between the back and segmentation points (β = −0.07, *SE* = 0.05, *z* = −1.56, *p* = 0.262), and also no significant (inverse) RT difference between the back and front points (β = −0.07, *SE* = 0.05, *z* = −1.47, *p* = 0.305). That is to say, the CLL group has a level RT pattern at event boundaries, as shown in Figure 4C.

**Figure 6:**
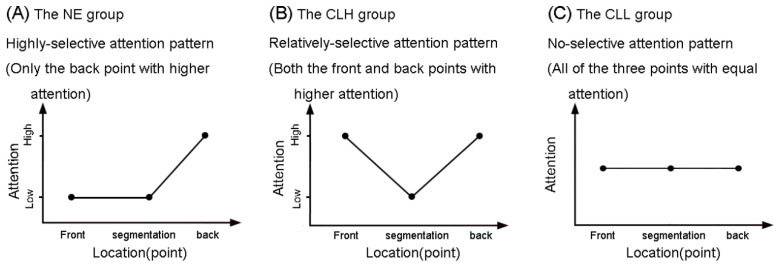
Schematic illustration of the NE, CLH, and CLL groups’ attention patterns at segmentation boundaries. Note. NE: the native English group; CLH: Chinese learners of English with relatively higher language proficiency; CLL: Chinese learners of English with relatively lower language proficiency.

In consideration of the negative correlation between RT and attention, as discussed in the Methodology [60,61], the three groups’ RT patterns were transformed into their respective attention pattern, as in Figure 6. As we can see, the NE, CLH, and CLL groups showed different attention patterns at segmentation boundaries. This actually answers the question whether Chinese learners of English (the CLH and CLL groups) performed a normative perceptual segmentation of English continuous speech, just as the native (the NE group), and the answer is no, because they did not show the same temporally selective attention pattern to that of the native, like the NE group in Figure 6. To better describe the difference of these three attention patterns, we introduce the concept of attention selectivity below.

Here, we tentatively define the selectivity of attention distribution as the proportion of the number of time points triggering relatively more attention relative to the whole number of time points, such that (1) the smaller the proportion, the higher the selectivity of attention distribution, and (2) 0 and 1 are the extreme limits, which signify no selectivity in the attention distribution. As for another aspect for selectivity, i.e., how much the higher attention (e.g., to one point) supersedes the lower attention to (e.g., to another point), it is not the primary concern in this study so will not be discussed here.

Given the tentative definition for attention selectivity, as shown in Figure 6, the NE group showed a highly selective attention pattern (Figure 6A), triggering more attention only at the back point (than the front point and the segmentation point); the CLH group had a relatively selective attention pattern (Figure 6B), distributing more attention to both the front and back points (than the segmentation point), and the CLL group revealed a non-selective attention pattern (Figure 6C), triggering equal attention to all three time points. Therefore, it can be concluded that the higher people’s language proficiency level becomes, the higher selectivity is shown in attention distribution at segmentation boundaries.

**Figure 7:**
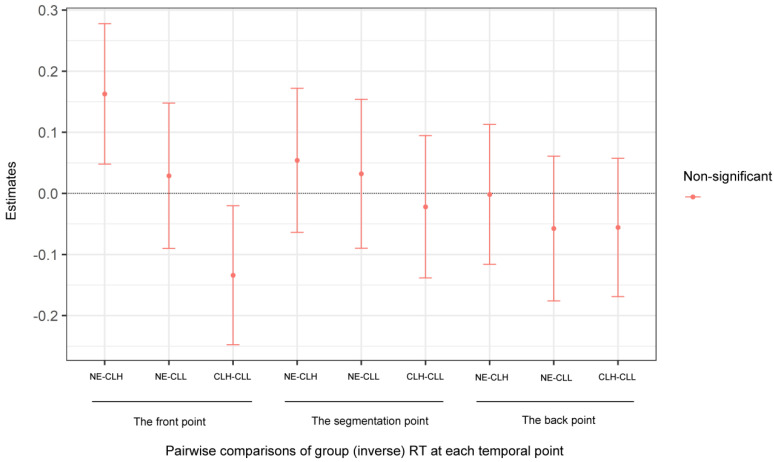
Pairwise comparisons of the three groups (the NE, CLH, and CLL groups)’ inverse RT values at each of the specific temporal points (the front, segmentation, and back points). Note. RT: response time; NE: the native English group; CLH: Chinese learners of English with relatively higher language proficiency; CLL: Chinese learners of English with relatively lower language proficiency.

Furthermore, as mentioned before, LMM on the (inverse) RT revealed a non-significant main effect of Group (χ^2^ (2) = 0.67, *p* = 0.716), which indicates that the NE, CLH, and CLL groups actually triggered the same amount of time at event boundaries. Additionally, post hoc pairwise comparisons of the (inverse) RT for the three groups at each specific time point also showed that there was no (inverse) RT difference among the NE, CLH, and CLL groups, whether at the front point, the segmentation point, or the back point, as displayed in Figure 7. This further proves the importance of distributing attention effectively. Specifically, it is how to distribute attention effectively (just like the NE group) rather than the overall amount of attention at the event boundary, and also rather than the amount of attention at each specific temporal point that enables a proper perceptual segmentation.

### 3.2. Recognition Memory

**Figure 8:**
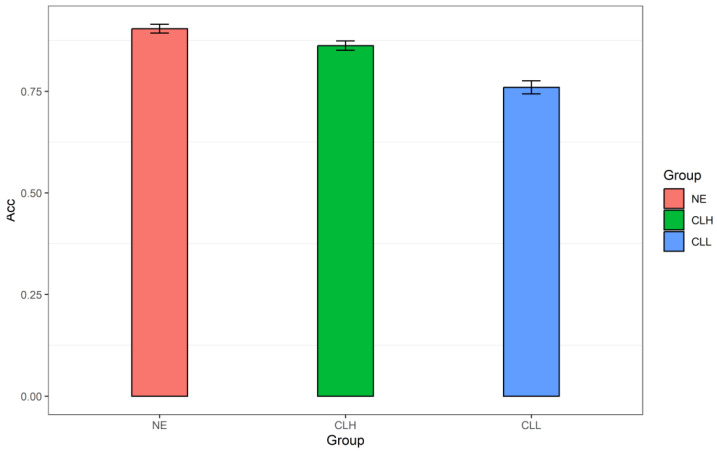
The mean proportion correct of the NE, CLH, and CLL groups in the recognition memory test. Note. Acc: recognition accuracy; NE: the native English group; CLH: Chinese learners of English with relatively higher language proficiency; CLL: Chinese learners of English with relatively lower language proficiency.

As for the recognition memory test, all of the CLH participants achieved a higher test accuracy than the chance level (0.5); two participants in the CLL group had lower accuracy than the chance level, i.e., 0.43 and 0.47, respectively, whose data were trimmed. There were none in both the CLH and CLL groups failing to answer in the recognition test. Finally, as shown in Figure 8, the correct mean proportion in the CLH group was 0.86 (*SD* = 0.35); the CLL group’s correct mean proportion was 0.74 (*SD* = 0.44). In order to compare, the corresponding data of the NE group in the recognition test (*M* = 0.90, *SD* = 0.30) were also included here.

GLMM on the binary response in the recognition test (‘0’ and ‘1’) showed a significant main effect of Group (χ^2^ (2) = 15.22, *p* < 0.001). Using the “emmeans” package, post hoc pairwise comparisons among the three groups were performed. The results revealed that the NE group had higher recognition accuracy than the CLL group (β = 1.31, *SE* = 0.35, *z* = 3.69, *p* = 0.001). The CLH group also performed with higher accuracy than the CLL group (β = 0.99, *SE* = 0.34, *z* = 2.94, *p* = 0.009). Nevertheless, there was no significant difference between the NE and CLH groups (β = 0.32, *SE* = 0.35, *z* = 0.93, *p* = 0.622). Therefore, in regard to the recognition memory, the CLH group did as well as the NE group, both of which did better than the CLL group (NE = CLH > CLL).

### 3.3. Correlation Between the Attention at Event Boundaries and Subsequent Recognition Memory

**Figure 9:**
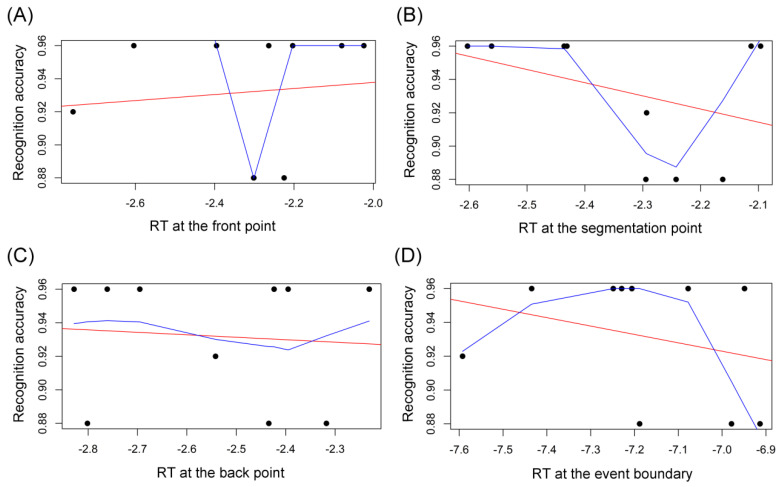
The NE group’s correlation relationships between RT and recognition memory. (**A**) The NE group’s correlation relationships of the RT at the front point and the subsequent recognition memory. (**B**) The NE group’s correlation relationships of the RT at the segmentation point and the subsequent recognition memory. (**C**) The NE group’s correlation relationships of the RT at the back point and the subsequent recognition memory. (**D**) The NE group’s correlation relationships of the RT at the whole event boundary and the subsequent recognition memory. Note. RT: response time. The red line: regression line. The blue line: lowess line.

As for the NE group, there was no outlier in both the RT data (including the summed RT across the three points, and the RT at each time point) and the data of recognition accuracy. As shown in Figure 9 and Table 1, Pearson’s r correlation analysis disclosed that there was no correlation between the RT at each of the three time points and the recognition accuracy: the correlation between the RT for the front point and the recognition accuracy (*r* = 0.11, *p* = 0.768; Figure 9A); the correlation between the RT for the segmentation point and the recognition accuracy (*r* = −0.37, *p* = 0.288; Figure 9B); and the correlation between the RT for the back point and the recognition accuracy (*r* = −0.08, *p* = 0.819; Figure 9C). In addition, there was no significant correlation between the total RT at the event boundary (summing the RT at the front, segmentation, and back points) and the subsequent mean proportion correct in the recognition memory test (*r* = −0.28, *p* = 0.430; Figure 9D). Given that the attention distribution at boundaries was modulated by perceptual segmentation [5], the lack of correlation between the attention at segmenting boundaries and later memory for the NE group means that perceptual segmentation did not play a role in the NE group’s subsequent recognition memory.

Given the negative relationship between RT and attention, as shown above [60,61], for simplicity, Table 1 is transformed into Table 2, below, so as to clearly reveal the relationship between the attention at the event boundary and the recognition memory.

As for the CLH group, there was no outlier in the recognition accuracy data and also no outlier in the total RT data at event boundaries (summing the RT values at the front, segmentation, and back points). When checking the RT data at each specific point, there was no outlier at the front and segmentation points but one outlier at the back point. As for the outlier sentence, both its RT data and corresponding recognition accuracy data were dropped when calculating the correlation.

**Figure 10:**
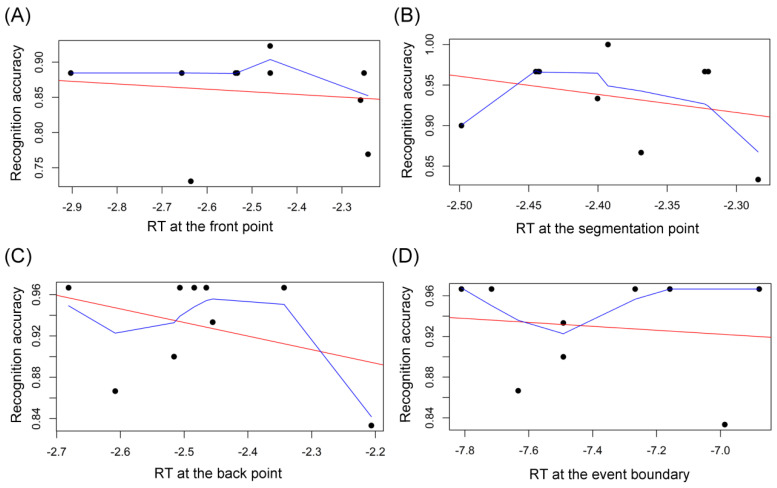
The CLH group’s correlation relationships between RT and recognition memory. (**A**) The CLH group’s correlation relationships of the RT at the front point and the subsequent recognition memory. (**B**) The CLH group’s correlation relationships of the RT at the segmentation point and the subsequent recognition memory. (**C**) The CLH group’s correlation relationships of the RT at the back point and the subsequent recognition memory. (**D**) The CLH group’s correlation relationships of the RT at the whole event boundary and the subsequent recognition memory. Note. RT: response time. The red line: regression line. The blue line: lowess line.

As presented in Figure 10 and Table 1, Pearson’s r correlation analysis discovered that there was no correlation between the RT at each of the three time points and the later-on mean proportion correct in the recognition: no correlation between the RT at the front point and the recognition accuracy (*r* = −0.01, *p* = 0.986; Figure 10A); no association between the RT at the segmentation point and the recognition accuracy (*r* = −0.29, *p* = 0.424; Figure 10B); and no correlation between the RT at the back point and the recognition accuracy (*r* = −0.36, *p* = 0.348; Figure 10C). Also, there was no significant association between the total RT at the event boundary and the subsequent mean proportion correct in the recognition memory test (*r* = −0.12, *p* = 0.752; Figure 10D). Accordingly, the relationship between the CLH group’s attention at the event boundary and the recognition memory is shown in Table 2. As mentioned above, this shortage of correlation between the CLH group’s attention at segmenting boundaries and later memory indicates that the CLH group’s (non-normative) perceptual segmentation also does not have an influence on later-on recognition memory for sentences.

**Figure 11:**
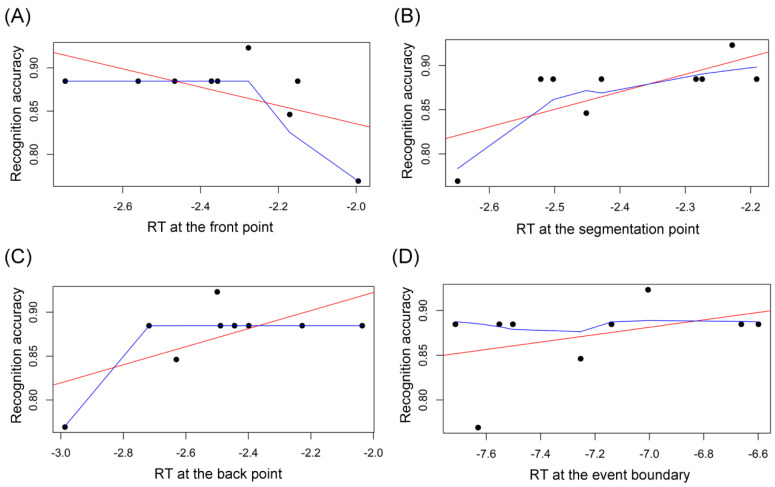
The CLL group’s correlation relationships between RT and recognition memory. (**A**) The CLL group’s correlation relationships of the RT at the front point and the subsequent recognition memory. (**B**) The CLL group’s correlation relationships of the RT at the segmentation point and the subsequent recognition memory. (**C**) The CLL group’s correlation relationships of the RT at the back point and the subsequent recognition memory. (**D**) The CLL group’s correlation relationships of the RT at the whole event boundary and the subsequent recognition memory. Note. RT: response time. The red line: regression line. The blue line: lowess line.

As for the CLL group, there was one outlier in the recognition accuracy, whose data of both RT and recognition accuracy were removed. As shown in Figure 11, Pearson’s r correlation analysis revealed that there was no significant correlation between the RT at the front point and the following recognition accuracy (*r* = −0.56, *p* = 0.114; Figure 11A). However, there was significant correlation between the RT at the segmentation point and the recognition accuracy (*r* = 0.72, *p* = 0.030; Figure 11B) and also marginally significant correlation between the RT at the back point and the recognition accuracy (*r* = 0.66, *p* = 0.055; Figure 11C). Finally, there was no correlation relationship between the total RT for the event boundary and the subsequent recognition accuracy (*r* = 0.40, *p* = 0.293; Figure 11D). Accordingly, the relationship between the CLL group’s attention at the event boundary and the recognition memory is displayed in Table 2. That is, for the CLL group, there are some correlations between the attention at (the segmentation and back points of) segmenting boundaries and recognition memory. This suggests that the CLL group’s (non-normative) perceptual segmentation may predict later memory for sentences. At first glance, it may seem surprising why the CLL group’s non-normative or improper perceptual segmentation may predict subsequent memory. There are two reasons to account for this, as follows: first, it is the coarse-grained (phrasal) segmentation boundaries that were used in this experiment, because the boundary had been identified to ask native English-speaking listeners to segment each audio sentence in as large units as possible, as shown in the Methodology. That indicates that the NE group’s highly selective attention pattern is accompanied with the coarse-grained segmentation. Thus, to be more exact, the NE group performed a normative coarse segmentation. And the CLL group’s non-selective attention pattern implies that the CLL group performed a non-normative coarse-grained segmentation. Perceptual segmentation actually undergoes multiple time scales simultaneously, i.e., finer segmentation is recursively enclosed by coarser segmentation, producing hierarchical structure [20,50] It is most likely that the CLL group performed a finer segmentation, such as shorter-phrase segmentation or word segmentation. Therefore, it is at finer segmentation boundaries that the CLL group’s attention to segmentation and back points predict later memory. Indeed, a previous study proved that less-skilled segmentation is inclined to have segmentation agreement at the finer grain rather than the coarser grain [20]. Second, the CLL group showed the lowest language proficiency in the three groups, which means it is the least possible for the CLL group to lean on language knowledge to decipher the audio sentences. At this time, the CLL group had to rely on the structure yielded from perceptual segmentation to try to memorize the sentence [30,38]. This accounts for the CLL group’s correlation between attention at boundaries and subsequent memory for sentences.

Intriguingly, as we can see from Figure 9, Figure 10 and Figure 11, the CLL group’s numerical trend (of the correlation between the attention at segmentation boundaries and recognition memory) is quite different from that of the NE group’s; that of the CLH group’s is in between. This difference is matched with their respective language proficiency. The reason may be relevant to the different perceptual segmentation (coarser or finer-grained) performed by the three groups, as discussed above.

Last, it should be of note that, until now, these findings of attention patterns at segmentation boundaries, and their prediction of later memory, should be cautiously confined to young healthy adults’ perceptual segmentation, because aged adults and people with Schizophrenia, Obsessive-Compulsive Disorder, Parkinson’s Disease, Lesions of the Prefrontal Cortex, and Alzheimer’s Disease are shown to have different perceptual segmentation from the sample recruited in this experiment [20].

In sum, the results firstly showed that Chinese learners of English (the CLH and CLL groups) had a different attention pattern at segmenting boundaries of continuous English speech from that of the native’s (the NE group’s). Specifically, in the sense of attention selectivity, the NE showed a highly selective attention pattern, while the CLL group had a non-selective attention pattern; the CLH group was in between, with a relatively selective attention pattern. This corresponds to their different levels of language proficiency: the NE group are native English-speaking listeners with high language proficiency, while the CLL group are Chinese learners of English with the lowest language proficiency (in the three groups); the CLH group’s language proficiency is in between. Given that the NE group’s highly selective attention pattern is together with normative perceptual segmentation, it indicates both the CLH and CLL groups segmented English speech stream in a non-normative manner, thereby displaying the role of language proficiency in perceptual segmentation. This testified the first hypothesis, that language proficiency can affect the attention pattern at segmenting boundaries and perceptual segmentation.

In addition, the experimental results disclosed that the NE and CLH groups had higher recognition accuracy than the CLL group. Because the NE and CLH groups have higher language proficiency than the CLL group, it suggests the effect of language proficiency on recognition memory. In respect to the equal recognition accuracy between the NE and CLH groups, detailed explanation will be made in the Discussion part later.

Thirdly, the results also revealed that both the NE and CLH groups’ attention at boundaries did not correlate with their subsequent memory for audio sentences, while the CLL group’s attention at (the segmentation and back points of) boundaries predicted later memory. As mentioned above, both the NE and CLH groups have higher language proficiency than the CLL group. Therefore, perhaps the reason why the NE and CLH groups did not correlate their attention at boundaries with later memory was that they had higher language proficiency, i.e., enough knowledge about English language to facilitate memory independently. And the reason why the CLL group correlated their attention at boundaries was that they had lower language proficiency, that is, not sufficient knowledge on the English language to help their memory for audio sentences, so they had to rely on (the structure yielded from) perceptual segmentation to construct memory for sentences. This testifies the second hypothesis, that language proficiency seems to have a superior role than perceptual segmentation; perceptual segmentation would come next to make an impact on the subsequent memory if people’s language proficiency is low enough.

Certainly, there may be some limitations or factors unable to be strictly controlled in this experiment. First, there are, after all, cultural and language difference between Chinese learners of English (the CLH and CLL groups) and natives (the NE group). But we believe that this would not dominate the segmentation of English continuous speech given that there is also difference in attention, memory, and their correlations between the two Chinese learners of English with different language proficiencies. Second, because participants in the CLH group are English majors, and they are usually required to learn another language (such as Japanese, French, etc.) for about two years in their undergraduate studies in China, this might influence their attention pattern or correlation between attention at boundaries and later memory. In the NE group, there two participants are from the UK, and the rest are from the USA, so a sample from the same country might be better. Third, considering that the recognition memory test is a judgement about whether the visually presented written sentences are congruent with what has been heard, potential biases should have been further controlled for. As for the data, the entire sample size could be enlarged. In addition, as for the data analysis of correlation, because there are only 10 visually presented sentences, which are heard before as designed in the recognition memory test, this forced the correlation between attention at segmentation boundaries, and memory could only be analyzed using the data of these 10 sentences. If more sample and sentence data are available, maybe the CLL group’s attention at the front point of boundaries could also predict the later memory. Last, the behavioral method using the RT to reflect the attention in speech perception could be complemented by using other means, such as ERP, fMRI (functional magnetic resonance imaging), etc.

## 4. Discussion

There are two research questions in this study: First, does English language proficiency affect attention patterns at segmenting boundaries of English speech streams, thereby influencing perceptual segmentation? Second, what is the relationship between English language proficiency, perceptual segmentation, and subsequent memory? Accordingly, two hypotheses were outlined: First, English language proficiency enables one to make an impact on attention patterns at segmenting boundaries of English speech streams and perceptual segmentation. Second, English proficiency has priority in affecting subsequent memory for English audio sentences relative to perceptual segmentation; however, when English proficiency lowers, perceptual segmentation begins to play its role in later memory for these English sentences.

Thus, the first aim of this study was to explore the effect of language proficiency on the attention pattern at event boundaries of auditory English sentences, by checking whether Chinese leaners of English (the CLH and CLL groups) have a different attention pattern from that of natives (the NE group). If the CLH and CLL groups really have a different pattern from the NE group, it implies language proficiency also influences their perceptual segmentation of this English continuous speech, because the NE group’s highly selective attention pattern at segmentation boundaries has been shown to coexist with a normative perceptual segmentation. And the second purpose of this research was to investigate their (English language proficiency, perceptual segmentation) relationship with memory for English audio sentences, by examining the effect of English language proficiency on later recognition test memory and the correlation between attention at segmentation boundaries and subsequent memory.

### 4.1. Effects of English Language Proficiency on the Attention Patterns at Segmentating Boundaries of Continuous English Speech

As mentioned in the Results section, English language proficiency in this study was shown to have effect on the attention patterns at segmenting boundaries of continuous English speech and perceptual segmentation of English speech streams. Similarly, Astheimer et al. [77] also revealed the role of language proficiency in temporally selective attention along continuous English speech. They disclosed that while native English listeners distributed temporally selective attention to word onsets during speech perception, Chinese learners of English did not show such selective attention. One difference is that Astheimer et al. [77] examined the attention distribution around word boundaries using the ERP, while our study explored the attention distribution around phrasal boundaries using the behavioral RT. However, both studies testified the temporally selective attention along continuous English speech perception. Therefore, this study can be an extension or complement to Astheimer and Sanders’ series of relevant studies of temporal selective attention as an effective and efficient listening strategy for the perception of English speech streams [77,78,79,80].

Though it has been testified that a highly selective attention pattern at segmentation boundaries coexists with the native English speakers’ normative perceptual segmentation in a prior study [6], the attention pattern at segmentation boundaries is so sensitive to changes in language proficiency, such as the CLH and CLL groups’ different attention patterns. Thus, it further testifies the potential of attention patterns at perceptual boundaries as another indicator to reflect perceptual segmentation. Future research could also probe into the issue about whether this attention pattern at perceptual boundaries is workable to be an indicator in different listening conditions, such as English speech stream with different speech intelligibility. To our knowledge, the exploration of an attention pattern at perceptual boundaries in the theoretical background of EST is minimal in the literature. Therefore, to some extent, this study contributes to this by breaking fresh ground in EST studies.

In addition, the present study displayed that the CLH group approximates the NE group more in the attention selectivity at event boundaries than the CLL group. In other words, the CLH group performed better perceptual segmentation than the CLL group, though both groups had a non-normative segmentation relative to natives. This also accords with Gilbert et al. [21]’s declaration that a higher language proficiency level is needed for second language learners of French to perform the phrasal parsing of English utterances like native English speakers. In this sense, this study supports the important role of language proficiency in perceptual segmentation by expanding this finding to Chinese learners of English. Meanwhile, this also affirmed the proposed effect of knowledge on perceptual segmentation in the EST [5]. Further, it extends relevant empirical practice on the function of knowledge on perceptual segmentation from an action sequence [23,38] or narratives [53] to isolated audio English sentences.

Regarding the debate on whether event segmentation is solely dependent on perceptual inputs, independent of knowledge [27,28], the present study cannot directly address this issue. This is because all three groups in the current study possess varying degrees of knowledge related to the English language. However, the CLH and CLL groups’ different attention patterns at segmenting boundaries in this study indeed implicate the effect of knowledge on perceptual segmentation. Maybe it is just as inferred in the Introduction, that language proficiency belongs to the type of knowledge achieved from long-term immersion into the object. Therefore, it may have reshaped the early perceptual processing like perceptual segmentation through mechanisms, such as attentional weighting, stimulus imprinting, differentiation, and unitization, just like that of the expertise [39].

### 4.2. Effect of English Language Proficiency and Perceptual Segmentation on Subsequent Memory

As shown by the results, both the NE and CLH groups had higher recognition accuracy than the CLL group in the recognition memory test, while the NE and CLH groups achieved the same recognition accuracy. As for the same memory performance of the NE and CLH groups, it is possibly because the recognition test adopted in this experiment was relatively easier in nature than the recall test [38,81]. Further, the tested sentences were verbatim originally heard sentences, which could provide more cues to participants for judgement than the paraphrased or referenced ones [82]. Thus, the recognition test in this study was probably not difficult enough to distinguish the memory ability between the NE and CLH groups.

Additionally, the results also disclosed that both the NE and CLH groups did not have correlation between their attention at boundaries and memory, while the CLL group did. As explained above in the Results, the reason might lie in that the NE and CLH groups had enough language knowledge to help construct memory independently (without resorting to perceptual segmentation), but the CLL group did not possess enough knowledge about English language so that structures produced from perceptual segmentation became the only cues to help enhance later memory. This is supported by prior studies [30,38]) showing that people will rely on the reconstruction of knowledge for better memory if relevant knowledge is accessible, but they will next resort to event segmentation to improve event memory when there is little relevant knowledge to count on. Further, because it is in the visual perception of action sequences that previous studies investigated the effect of knowledge and segmentation on subsequent memory, the finding extended this finding to the auditory perception of speech streams. However, in view that there is still one point (i.e., the front point) whose attention is not correlated with later memory for the CLL group, the reason may be the minimal data available for correlation analysis, as put forward in the Results, so further research is needed to strengthen this finding.

We can see that the results in this experiment seem to support the effect of knowledge on memory for events [7,8,57,58], as shown by the NE and CLH groups’ better memory than the CLL group, and also seem to endorse the role of segmentation on later-on memory [10,11], as revealed in the CLL group’s correlation between their attention at boundaries and memory. In other words, it seems to agree with the independent role of knowledge and segmentation in subsequent memory [4,56], if two factors are not comprehensively examined in one study. The point is that whether it is knowledge or segmentation that contributes to later memory seems to be dependent on people’s English language proficiency (the NE and CLH groups, or the CLL group). In a word, whether knowledge and segmentation make their independent effect on later-on memory depends on whether enough relevant knowledge is available.

Certainly, there is also an alternative interpretation that both knowledge and segmentation impact later memory simultaneously, though the CLL group had their attention correlation at boundaries with later memory. Indeed, this study cannot tease out the possibility that language proficiency also plays a role in the CLL group’s memory for sentences, because, after all, the CLL group has knowledge of the English language, though it is the lowest in this study. Hence, future research needs to take knowledge and segmentation into consideration comprehensively to study their effect on memory, on the one hand, and, on the other hand, to check whether language knowledge is still there to exert its influence on the memory of people with low language proficiency, or how low language proficiency leads to their null effect on memory.

## 5. Conclusions

First, among the several arguments regarding whether knowledge can affect perceptual segmentation, this study, in a novel way, affirms the role of knowledge in perceptual segmentation proposed by the EST, i.e., by showing the role of language proficiency in the NE group’s typical attention pattern at segmenting boundaries (coexisting with their normative perceptual segmentation). Second, among the several proposals in the literature about the effect of knowledge and perceptual segmentation on subsequent memory, the current study is inclined to endorse one of them: knowledge has a superior role on later memory than perceptual segmentation; however, perceptual segmentation indeed comes next to affect later-on memory when relevant knowledge is not or is insufficiently available. Previous studies have testified it in the visual perception of an action sequence, while our study extended this to the auditory perception of continuous speech, by showing that the NE and CLH groups had better recognition memory for English audio sentences than the CLL group, but the NE and CLH groups’ attention at segmenting boundaries of these audio sentences was not correlated with their recognition memory; the CLL group’s one predicted their later memory. As outlined in the previous literature, the mechanism by which knowledge and perceptual segmentation influence memory is to provide structures for memory construction, whether knowledge structures (brought by knowledge) or sequential and hierarchical structures (yielded from perceptual segmentation).

As for the implications of this study for language learning and teaching, firstly, the role of English language proficiency in perceptual segmentation implicates that, aside from intensive listening practice, increasing the general knowledge of English (such as lexis, grammar, pragmatics, etc.) may also be a way of improving the perception of English speech streams, given that perceptual segmentation facilitates the perception and comprehension of continuous speech. Secondly, the effect of English proficiency on subsequent memory also has implications that it could be an effective way to improve memory for English sentences by accumulating language knowledge. In addition, the association between perceptual segmentation and memory for the CLL group implies that if little knowledge is available, it might be a means to intentionally segment the sentences to help improve memory. As for the implications of this study for cognitive sciences, given that continuous speech perception cannot be performed with accuracy even by very powerful automatic speech recognition systems [83,84], the attention pattern at segmenting boundaries of continuous speech might provide some hints for improving AI (artificial intelligence)’s perception of natural language by imitating human being’s attention pattern to facilitate perceptual segmentation. Additionally, the effect of knowledge on perceptual segmentation in this study indicates that feeding enough knowledge data into AI is also a way to increase perceptual accuracy for continuous speech perception.

This study is mainly limited by the following factors: First, the relatively small entire sample size and sentence data possibly obstruct the appearance of some significant effect, for example, the correlation between the attention at the front point and later memory. Second, the CLH group generally has two-year learning experience of a third language in their undergraduate studies, which may be a confounding factor. Aside from making up the above-mentioned limitations, future research could further examine the reliable coexistence of the NE group’s normative segmentation and their highly selective attention pattern at boundaries by checking whether this attention pattern is influenced by the speech intelligibility of English continuous speech. In addition, other sample learners of English who are not Chinese could also be employed to generalize the relevant findings in this study.

## Figures and Tables

**Table 1:** Correlation coefficient (*p* value) between the three groups (the NE, CLH, and CLL groups)’ RT at the event boundary (including the RT at each of the three points and the total RT summing the RT at each point) and the recognition accuracy.

	Front RT	Segmentation RT	Back RT	Total RT
NE recognition accuracy	0.11 (0.768)	−0.37 (0.288)	−0.08 (0.819)	−0.28 (0.430)
CLH recognition accuracy	−0.01 (0.986)	−0.29 (0.424)	−0.36 (0.348)	−0.12 (0.742)
CLL recognition accuracy	−0.56 (0.114)	0.72 (0.030) *	0.66 (0.055) ^+^	0.40 (0.293)

Note. RT: response time; Front RT: RT at the front point; Segmentation RT: RT at the segmentation point; Back RT: RT at the back point; Total RT: total RT summing the RT at the front, segmentation, and back points; NE: the native English group; CLH: Chinese learners of English with relatively higher language proficiency; CLL: Chinese learners of English with relatively lower language proficiency. * *p* < 0.05; ^+^ *p* < 0.1.

**Table 2:** Correlation coefficient (*p* value) between the three groups (the NE, CLH, and CLL groups)’ attention at the event boundary (including the attention at each of the three points and the total attention summing the attention at each point) and the recognition memory.

	Front Attention	Segmentation Attention	Back Attention	Total Attention
NE recognition memory	−0.11 (0.768)	0.37 (0.288)	0.08 (0.819)	0.28 (0.430)
CLH recognition memory	0.01 (0.986)	0.29 (0.424)	0.36 (0.348)	0.12 (0.742)
CLL recognition memory	0.56 (0.114)	−0.72 (0.030) *	−0.66 (0.055) ^+^	−0.40 (0.293)

Note. Front attention: attention at the front point; Segmentation attention: attention at the segmentation point; Back attention: attention at the back point; Total attention: total attention at the event boundary; NE: the native English group; CLH: Chinese learners of English with relatively higher language proficiency; CLL: Chinese learners of English with relatively lower language proficiency. * *p* < 0.05; ^+^ *p* < 0.1.

## Data Availability

The original data presented in the study are openly available in https://osf.io/khtq4/ (accessed on 24 November 2024).
